# From Biomarker Measurement to Clinical Utility: Lessons From Six Established Biomarkers

**DOI:** 10.7759/cureus.117934

**Published:** 2026-10-14

**Authors:** Elsa Faviou, Christina Economidou, Melina E Baltogianni, Nikolaos Baltogiannis

**Affiliations:** 1 Biopathology and Laboratory Medicine, Affidea of Athens, Athens, GRC; 2 Obstetrics and Gynecology, Patras General Hospital "Agios Andreas", Patras, GRC; 3 Pediatric Surgery, Private Children’s Hospital of “Athens Medical Group”, Athens, GRC

**Keywords:** biomarkers, clinical decision-making, clinical utility, diagnostic testing, laboratory medicine, precision medicine, surrogate endpoints, value-based healthcare

## Abstract

Biomarkers are increasingly used to support diagnosis, risk stratification, therapeutic selection, monitoring, and prognosis. Advances in high-sensitivity assays, molecular diagnostics, multi-omics, and artificial intelligence have accelerated biomarker discovery, but improved measurement does not necessarily translate into improved care. A biomarker may be analytically robust and strongly associated with disease while adding little to clinical decision-making. This narrative review examines the progression from analytical validity and clinical association to incremental value, actionability, clinical utility, and net clinical benefit. Six familiar biomarkers were selected as illustrative case studies because each represents a different point at which biomarker translation may succeed or fail. D-dimer shows how clinical context and pretest probability can transform a nonspecific marker into an efficient diagnostic tool. Procalcitonin demonstrates that utility often depends on the management algorithm attached to the result. High-sensitivity cardiac troponin illustrates both the benefits and the interpretive burden created by detecting smaller degrees of myocardial injury. Point-of-care C-reactive protein testing shows that clinical value does not require technological novelty. Conversely, N-terminal pro-B-type natriuretic peptide demonstrates that strong prognostic value does not necessarily validate biomarker-targeted therapy, while cancer antigen 125 screening for ovarian cancer shows that earlier detection and stage shift cannot be assumed to improve mortality and may expose patients to downstream harm. Taken together, these examples suggest that the clinically relevant unit is not the biomarker in isolation but the biomarker-enabled decision pathway. Biomarker research should therefore move beyond association and discrimination toward evidence that testing changes meaningful decisions and that the resulting strategy provides greater benefit than harm compared with current care.

## Introduction and background

Modern medicine has become exceptionally good at measuring biology. A biomarker is a measurable biological characteristic used to indicate a normal biological process, a disease process, or a response to an intervention. High-sensitivity assays and rapidly evolving molecular technologies continue to expand the number and complexity of candidate biomarkers available for clinical investigation [1-4]. The more difficult question is what to do with this information once it has been generated.

A biomarker may be measured with excellent precision, differ significantly between patients with and without disease, remain independently associated with an outcome after multi-variable adjustment, and improve the discrimination of a predictive model. None of these findings alone establishes that measuring the biomarker will improve patient care [5-7].

Analytical validity refers to whether an assay measures the biomarker accurately and reproducibly, whereas clinical validity refers to whether the biomarker is associated with the clinical condition or outcome of interest. Discrimination describes how well a marker distinguishes between patients with and without an outcome, while reclassification asks whether adding the marker moves patients into different clinically meaningful risk groups. Incremental value is the information gained beyond existing clinical assessment. Actionability means that the result can change a clinical decision; clinical utility asks whether using the test improves care; and net clinical value weighs the resulting benefits against harms, burdens, and costs [5-7].

This gap is not merely theoretical. Better detection, biomarker-guided treatment, or earlier diagnosis may appear promising while failing to improve the outcome they were expected to influence, as shown by randomized evaluations of high-sensitivity troponin implementation, natriuretic peptide-guided therapy, and ovarian cancer screening [8-10].

The methodological literature has long recognized this distinction. Pletcher and Pignone argued that biomarker evaluation should ultimately address health impact rather than stop at predictive performance [5]. Bossuyt et al. similarly distinguished diagnostic accuracy from clinical utility, emphasizing that the relevant question is whether using a test improves outcomes compared with the best available alternative strategy [6]. Moons et al. subsequently focused on incremental value as follows: before adopting a new biomarker, one should establish what information it adds to what is already known from clinical assessment and existing investigations [7].

Progression through these stages is not automatic. A statistically significant biomarker may add little to individual prediction, a prognostic marker may identify a high-risk group for which no effective intervention exists, and an actionable result may trigger an intervention that fails to improve outcomes. Screening may detect disease earlier while exposing healthy individuals to false-positive findings and unnecessary procedures. For this reason, actionability and net clinical value require evidence beyond the analytical and association studies used during biomarker discovery. The opposite is also possible. An imperfect and biologically nonspecific biomarker may be highly valuable if it reliably resolves uncertainty at a common clinical decision point.

A further concept is essential - context of use. A biomarker is never simply “useful” or “not useful” in the abstract. Its evidentiary requirements depend on the role it is expected to play. A marker used to exclude pulmonary embolism, guide antibiotic discontinuation, estimate prognosis, enrich a clinical trial population, or screen healthy individuals answers a different clinical question in each setting. The same biomarker may therefore have very different utility depending on the intended decision.

A prognostic biomarker estimates the likelihood of a future outcome, whereas a predictive biomarker identifies whether the effect of a specific intervention is likely to differ according to biomarker status. A surrogate endpoint is different as follows: it is used in place of a patient-important outcome and requires evidence that treatment effects on the biomarker reliably reflect treatment effects on the clinical outcome [4]. These roles should not be used interchangeably.

This review builds on the methodological frameworks of Pletcher and Pignone, Bossuyt et al., and Moons et al. by bringing their principles together in a single biomarker-enabled decision pathway [5-7]. It does not propose a new taxonomy or rank biomarkers by diagnostic performance. Instead, it examines where translation from measurement to patient benefit can succeed or fail. The six biomarkers were selected because they have mature evidence bases, represent different clinical uses, and illustrate distinct translational patterns as follows: context-dependent diagnostic use, algorithm-guided treatment, improved detection without assured outcome benefit, prognostic information without a validated treatment target, and screening based on an intermediate endpoint. The pathway connects measurement, interpretation, decision, intervention, and outcome within one practical framework.

## Review

Literature search and approach

We conducted a targeted narrative literature search in PubMed/MEDLINE through September 3, 2026. Search concepts included combinations of “biomarker,” “clinical utility,” “clinical validity,” “incremental value,” “diagnostic testing,” “clinical decision-making,” “decision curve,” “net benefit,” “surrogate endpoint,” and “value-based laboratory medicine,” together with biomarker-specific terms relating to D-dimer, procalcitonin, high-sensitivity cardiac troponin, C-reactive protein (CRP), N-terminal pro-B-type natriuretic peptide (NT-proBNP), and cancer antigen 125 (CA-125). Reference lists of key methodological papers, systematic reviews, and pivotal trials were also reviewed.

Studies were selected for detailed discussion when they evaluated the effect of biomarker testing on clinical decisions, management, resource use, patient-important outcomes, or the methodological assessment of clinical utility. Priority was given to prospective management studies, randomized controlled trials, systematic reviews and meta-analyses, and methodological papers. Studies limited to biomarker discovery, analytical performance, or biomarker-outcome association without a clear link to clinical decision-making were used only when required to provide conceptual background.

When several studies addressed the same question, emphasis was placed on pivotal prospective trials and the most informative contemporary systematic reviews. Where findings differed across studies or strategies, the disagreement was retained rather than resolved by selective citation.

The six biomarkers were not selected through formal ranking or exhaustive screening. They were chosen because each represents a distinct stage or failure mode in the pathway from measurement to clinical utility and because substantial prospective evidence is available for each. Selection therefore prioritized biomarkers with well-documented translational trajectories rather than citation count, novelty, or publication recency.

Evidence was synthesized narratively by examining where each biomarker progressed or failed across analytical validity, clinical validity, incremental value, actionability, clinical utility, and net clinical value. No original meta-analysis, meta-regression, or recalculation of published estimates was performed. Quantitative pooling across the six case studies was not considered appropriate because the biomarkers differed substantially in intended use, patient population, study design, comparator, intervention, and outcome. Effect estimates and measures of uncertainty were therefore reported from the original trials and meta-analyses. No formal duplicate screening, prospective record count, or study-level risk-of-bias scoring was performed.

From analytical validity to clinical utility

Analytical and clinical validity are necessary starting points, but they do not demonstrate that a test changes management or improves outcomes. A statistically independent predictor may add little to individual prediction, and improvements in discrimination or reclassification may not move patients across a threshold at which management changes [5-7].

Measures such as sensitivity, specificity, area under the receiver operating characteristic curve, calibration, and reclassification remain important, but they do not incorporate the clinical consequences of false-positive and false-negative classifications. A modest improvement may be valuable when it resolves uncertainty around an important treatment threshold, whereas a larger improvement may have little practical consequence if management remains unchanged.

Decision curve analysis addresses part of this limitation by estimating net benefit across clinically relevant threshold probabilities [11]. The underlying principle is straightforward as follows: the value of a prediction depends not only on its statistical performance, but also on whether the result changes a decision and whether the resulting strategy is better than current care.

The biomarker-enabled decision pathway

The six examples considered here ultimately converge on the same idea - the biomarker itself may be the wrong unit of analysis. The clinically relevant unit is the biomarker-enabled decision pathway (Figure 1). Failure can occur at any transition in the pathway. Consequently, claims of utility should identify where the supporting evidence begins and ends, and whether benefit has been demonstrated for the complete testing strategy.

**Figure 1:**
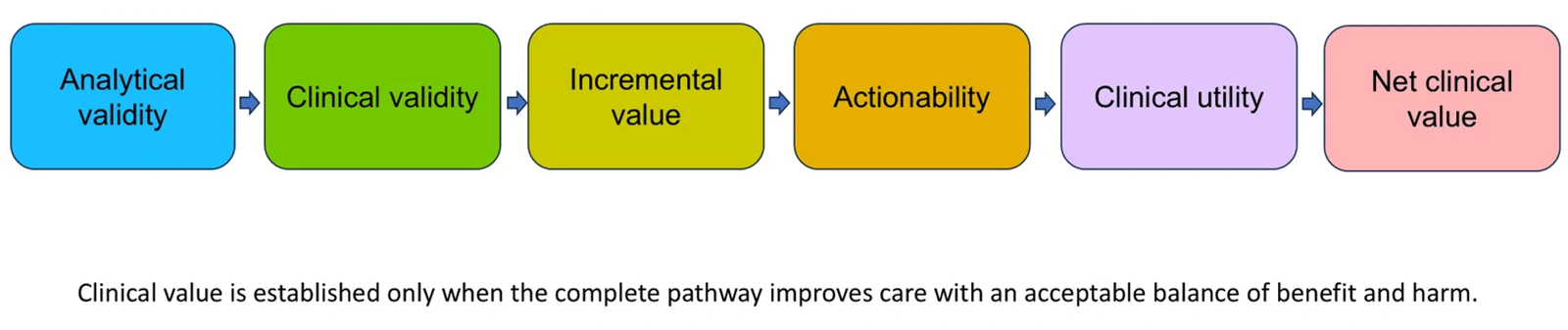
From biomarker to bedside decision - the biomarker-enabled decision pathway. This image was created manually by the authors (Elsa Faviou, Christina Oikonomidou, Melina E. Baltogianni, and Nikolaos Baltogiannis) of this study using Microsoft PowerPoint (Redmond, WA: Microsoft Corp.).

D-dimer: when context creates value

D-dimer is an instructive starting point because its limitations are well known. Although highly sensitive for venous thromboembolism, it has limited specificity and may be elevated with increasing age, inflammation, infection, malignancy, pregnancy, trauma, surgery, and many other conditions. Used indiscriminately with a fixed threshold, it generates a considerable number of positive results in patients who do not have pulmonary embolism. Its clinical value improved not because the biomarker itself changed, but because its interpretation became more sophisticated.

The Age-Adjusted D-Dimer Cutoff Levels to Rule Out Pulmonary Embolism (ADJUST-PE) study prospectively evaluated an age-adjusted D-dimer threshold in patients with suspected pulmonary embolism. In patients aged 50 years or older, the cutoff was defined as age multiplied by 10 rather than applying a conventional fixed threshold to everyone. Among patients with a D-dimer concentration above 500 μg/L but below the age-adjusted threshold, the three-month failure rate was 0.3% (95% CI: 0.1%-1.7%). Among patients aged 75 years or older, the proportion in whom pulmonary embolism could be excluded using D-dimer increased from 6.4% to 29.7%, without additional false-negative findings [12].

The YEARS study moved further toward probability-adapted interpretation. Three clinical criteria were combined with different D-dimer thresholds, allowing pulmonary embolism to be excluded in more patients without computed tomography pulmonary angiography (CTPA) [13].

The Pulmonary Embolism Graduated D-Dimer (PEGeD) study tested another clinical probability-adjusted approach. Pulmonary embolism was considered excluded in patients with low clinical pretest probability and a D-dimer below 1000 ng/mL, and in those with moderate probability and a D-dimer below 500 ng/mL. Imaging was required in 34.3% of patients under this strategy compared with an estimated 51.9% if a conventional threshold had been applied [14].

The broader evidence supports the same principle. An individual-patient-data meta-analysis of six prospective studies involving 7,268 patients found that combining the Wells rule with age-adjusted D-dimer testing increased the proportion of patients in whom imaging could be withheld from 28% to 33%, with a low three-month failure rate across the examined subgroups. Importantly, efficiency varied by clinical setting and patient characteristics, reinforcing that the value of D-dimer depends on the probability framework in which it is used rather than on a threshold applied in isolation [15].

These strategies should not be regarded as interchangeable thresholds that can be applied independently of their validated algorithms. Their safety depends on the clinical probability framework in which each was tested. The important change across these studies was conceptual rather than analytical. D-dimer ceased to function as a simple positive-or-negative test and became part of a probability-based decision pathway. Its value lies in safely avoiding imaging in selected patients, not in maximizing the number of abnormal laboratory results detected. The meaning of a biomarker therefore depends on the clinical context in which it is measured.

Procalcitonin: a result is only as useful as the decision attached to it

Procalcitonin (PCT) takes the argument one step further. Its clinical relevance is not simply that concentrations may rise in bacterial infection. What matters is whether measurement can improve antibiotic decisions. An individual patient-level meta-analysis of randomized ICU trials found that PCT-guided protocols reduced antibiotic exposure and were associated with lower mortality, although effects varied across populations and protocols [16].

A larger systematic review and meta-analysis published in 2023 included 26 randomized trials and 9,048 critically ill patients. PCT-guided therapy reduced antibiotic duration and was associated with a signal toward lower 28-day mortality, but recurrent infections were more frequent [17]. These findings argue against presenting PCT as a biomarker that simply “improves survival.”

The effect also depends on how the biomarker is used. Earlier systematic evidence showed that PCT-guided discontinuation can shorten antibiotic exposure, whereas strategies that used PCT to intensify antimicrobial therapy did not produce the same benefit and could increase treatment burden [18]. More recent evidence reinforces the stewardship signal. A 2024 network meta-analysis found that PCT-guided discontinuation shortened antibiotic treatment by 1.89 days compared with standard care (95% CI: -2.30 to -1.47 days) [19]. A 2026 rapid systematic review similarly found a mean reduction of 2.0 antibiotic days (95% CI: -2.6 to -1.4; I^2^=88.8%) and no clear reduction in mortality (RR: 0.95, 95% CI: 0.83-1.07) [20]. Differences in included populations, protocols, adherence, and outcome definitions contribute to the heterogeneity across these reviews. Taken together, the most reproducible benefit is reduced antibiotic exposure; any mortality advantage is less certain and should not be treated as the defining evidence of utility. The assay itself does not change between these strategies; what changes is the decision attached to the result. It is therefore misleading to speak of the “clinical utility of procalcitonin” without specifying the context of use. The intervention being evaluated is more accurately described as PCT-guided antibiotic management. More broadly, biomarker studies often need to evaluate measurement, interpretation, and the attached decision rule as a single clinical strategy.

High-sensitivity cardiac troponin: the benefits and limits of detecting more

High-sensitivity cardiac troponin assays illustrate what becomes possible, and harder to interpret, once analytical capability improves substantially. The ability to measure lower troponin concentrations and detect changes earlier has enabled accelerated diagnostic pathways for patients presenting with suspected acute coronary syndrome. In the High-Sensitivity Cardiac Troponin on Presentation to Rule Out Myocardial Infarction (HiSTORIC) stepped-wedge cluster randomized trial involving more than 31,000 patients, implementation of an early rule-out pathway using high-sensitivity cardiac troponin I reduced mean length of stay from 10.1 to 6.8 h and increased discharge from 50% to 71% [21]. These are meaningful clinical and operational gains. Resolving diagnostic uncertainty sooner matters to patients, reduces unnecessary observation, and releases hospital resources.

The safety result deserves careful interpretation. Although one-year outcomes were similar and there was no clear evidence of increased harm, the study did not formally meet its prespecified noninferiority criterion for the 30-day safety endpoint [21]. The operational benefit is convincing; the safety conclusion should remain appropriately measured.

The High-Sensitivity Troponin in the Evaluation of Patients With Acute Coronary Syndrome (High-STEACS) trial provides a complementary lesson. Implementation of a high-sensitivity cardiac troponin I assay reclassified 1,771 of 10,360 patients with myocardial injury. Only around one-third of those reclassified were ultimately considered to have type 1 myocardial infarction. Among reclassified patients, myocardial infarction or cardiovascular death at one year occurred in 15% during the validation phase and 12% during the implementation phase, without a significant difference (adjusted OR: 1.10, 95% CI: 0.75-1.61; p=0.62) [8].

This should not be interpreted as a failure of high-sensitivity troponin. Rather, it illustrates the interpretive responsibility created by improved analytical sensitivity. Troponin elevation is not synonymous with type 1 myocardial infarction. Myocardial injury occurs in heart failure, sepsis, kidney disease, pulmonary embolism, tachyarrhythmia, and numerous other conditions.

Greater analytical sensitivity therefore gives clinicians more biological information. Whether that information improves care depends on what the abnormality represents and whether an effective intervention follows, a reminder that better detection and better outcomes, while related, are not automatically the same thing.

The 2026 Mersey Acute Coronary Syndrome Rule-Out Study 2 (MACROS2) pragmatic randomized trial adds a further implementation lesson. Compared with a 0/3-h pathway, the ESC 0/1-h high-sensitivity troponin pathway was noninferior for safety among discharged patients but did not significantly increase discharge within 4 h. The investigators attributed much of the lost efficiency to central laboratory turnaround times and other system constraints [22]. Thus, even a validated biomarker algorithm may fail to deliver its expected operational benefit if the surrounding care pathway cannot support it.

C-reactive protein: useful does not have to mean new

The current biomarker landscape is understandably dominated by novelty as follows: multi-omics, circulating nucleic acids, complex molecular signatures, and artificial intelligence. CRP offers a useful counterpoint. It is old, inexpensive, and biologically nonspecific. Yet a biomarker does not have to be novel to solve a meaningful clinical problem.

A systematic review and meta-analysis of 13 studies involving 9,844 participants examined point-of-care CRP testing in patients with respiratory tract infections in primary care. Immediate antibiotic prescribing was reduced from 51.4% with usual care to 38.2% with CRP-guided assessment (RR: 0.79, 95% CI: 0.70-0.90; p=0.0003; I^2^=76%) [23]. Clinical recovery, symptom resolution, and hospital admission did not differ significantly.

However, there was a trade-off, as re-consultations within 30 days were more common with CRP point-of-care testing, 13.5% versus 9.7% [23]. That detail matters because utility should be assessed as net benefit rather than by selecting the most favorable endpoint. Earlier randomized evidence had similarly shown that CRP-assisted management could reduce antibiotic prescribing without impairing clinical recovery [24].

The updated 2022 Cochrane review provides broader support. Across 12 CRP point-of-care trials involving 10,218 participants, testing reduced antibiotic prescribing at the index consultation from an estimated 516 to 397 prescriptions per 1,000 patients (RR: 0.77, 95% CI: 0.69-0.86; I^2^=79%; moderate-certainty evidence) [25]. Yet the effect is not universal. In a 2024 French randomized trial of 404 children and adults, antibiotics were prescribed to 43.0% of patients in the CRP point-of-care group and 47.7% in the usual-care group (difference -4.7 percentage points, 95% CI: -14.4 to 5.0; p=0.30) [26]. The contrast is useful as follows: the same assay can have different practical effects when prescribing behavior, adherence to the decision rule, patient mix, and healthcare setting differ.

CRP does not diagnose every bacterial respiratory infection, nor does it need to. Its usefulness is more modest and practical as follows: it can reduce diagnostic uncertainty sufficiently to influence antibiotic prescribing in a setting where unnecessary treatment is common. An established marker used at the right decision point may therefore provide more clinical value than a technologically impressive new biomarker that does not change management.

NT-proBNP: predicting risk is not the same as treating it

Natriuretic peptides illustrate another common inferential step in biomarker medicine; they have well-established diagnostic and prognostic roles in heart failure. Higher concentrations are associated with disease severity and adverse outcomes. It is therefore intuitive to ask whether treatment specifically intensified until NT-proBNP falls below a target might improve prognosis.

The Guiding Evidence-Based Therapy Using Biomarker-Intensified Treatment in Heart Failure (GUIDE-IT) trial directly tested this hypothesis in patients with high-risk heart failure with reduced ejection fraction. Participants were randomized to an NT-proBNP-guided strategy targeting a concentration below 1000 pg/mL or to usual guideline-directed care. The trial stopped early for futility after 894 patients were enrolled. Heart failure hospitalization or cardiovascular death occurred in 37% of patients in both groups (adjusted HR: 0.98, 95% CI: 0.79-1.22; p=0.88) [9]. This does not justify a broad conclusion that all natriuretic peptide-guided strategies are ineffective in all heart failure populations. GUIDE-IT tested a particular strategy in a specific heart failure with reduced ejection fraction (HFrEF) population.

The wider trial literature is more nuanced than GUIDE-IT alone. A 2018 systematic review and individual-participant-data meta-analysis found that natriuretic peptide-guided therapy reduced heart failure hospitalization (HR: 0.81, 95% CI: 0.68-0.98) but not all-cause mortality (HR: 0.87, 95% CI: 0.75-1.01) or cardiovascular mortality (OR: 0.88, 95% CI: 0.67-1.16). Adverse events were more frequent with biomarker-guided therapy (OR: 1.29, 95% CI: 1.04-1.60), and treatment effects varied by age and ejection fraction [27]. More recently, a 2025 meta-analysis of nine randomized trials in acute decompensated heart failure found no significant reduction in all-cause mortality, cardiovascular death, or the composite of heart-failure hospitalization or cardiovascular death in the principal risk-ratio analyses [28]. These findings do not negate the prognostic value of natriuretic peptides; they show that prognostic strength does not automatically establish a universal therapeutic target. Its value for the present discussion is conceptual. A strong prognostic marker provides information about future risk, but it does not, by itself, establish that treatment directed toward changing the marker will improve outcome.

CA-125: earlier detection is not the final outcome

Ovarian cancer screening provides perhaps the clearest example of why an apparently favorable intermediate result cannot be assumed to represent clinical benefit. The reasoning behind screening is compelling. Advanced ovarian cancer carries a poor prognosis, so detecting more cancers at an earlier stage should, intuitively, reduce mortality. Randomized evidence shows why that assumption must be tested rather than accepted.

In the Prostate, Lung, Colorectal, and Ovarian (PLCO) Cancer Screening Trial, 78,216 women were randomized to annual screening with CA-125 and transvaginal ultrasound or usual care. Screening did not reduce ovarian cancer mortality. There were 118 ovarian cancer deaths in the screening group and 100 in the usual-care group, with no mortality benefit from screening (mortality RR: 1.18, 95% CI: 0.82-1.71) [29].

The downstream consequences were equally important. False-positive results led 1,080 women without ovarian cancer to undergo surgery, and 163 experienced at least one serious complication [29]. UK Collaborative Trial of Ovarian Cancer Screening (UKCTOCS) subsequently provided an even larger test of the early-detection hypothesis. More than 200,000 women were randomized to multi-modal screening incorporating longitudinal CA-125 interpretation, ultrasound screening, or no screening [30].

Long-term follow-up showed that multi-modal screening increased the incidence of stage I or II disease by 39.2% compared with no screening (95% CI: 16.1%-66.9%). However, after a median follow-up of 16.3 years, there was no significant reduction in ovarian or tubal cancer mortality (p=0.58) [10].

A subsequent comprehensive UKCTOCS assessment of mortality impact, risks, and benefits reached the same clinical conclusion. Multi-modal screening increased early-stage detection but did not reduce ovarian or tubal cancer deaths, and false-positive findings led to unnecessary surgery in women without cancer [31]. This is important because it evaluates the entire screening pathway rather than the analytical behavior of CA-125 alone. The finding is important because stage shift is intuitively persuasive. It looks like success. But the purpose of population screening was not simply to diagnose disease sooner; it was to improve outcomes.

Classic screening biases are relevant here. Lead-time bias can create the appearance of longer survival simply because diagnosis occurs earlier, even if the time of death is unchanged. Length bias favors detection of slower-growing disease with a longer preclinical phase and potentially better prognosis. Overdiagnosis may identify disease that would never have become clinically important during the patient's lifetime. These mechanisms help explain why earlier detection or improved stage distribution cannot be accepted as proof of benefit.

The problem extends beyond ovarian cancer and is directly relevant to multi-cancer early detection (MCED) testing. A methodological analysis of published screening trials across breast, lung, ovarian, and prostate cancer found that stage shift did not consistently predict the observed effect on disease-specific mortality [32]. Stage distribution may therefore be informative, but it should not be assumed to be a validated surrogate for mortality benefit.

Screening is offered largely to healthy people, among whom disease prevalence is low. False-positive findings can therefore produce serial testing, anxiety, invasive investigation, surgery, and complications in individuals who would never have benefited from the test. CA-125 screening thus exposes a central danger in biomarker evaluation as follows: an intermediate endpoint can improve while the outcome that matters most remains unchanged.

Biomarkers as surrogate endpoints

The NT-proBNP and CA-125 examples illustrate why surrogacy requires separate validation. An association between a biomarker and an outcome is not sufficient to establish a valid surrogate. Evidence is required that the effect of treatment on the biomarker reliably reflects its effect on the patient-important clinical outcome [33].

Biomarkers may still be valuable for diagnosis, risk stratification, treatment selection, monitoring, and safety assessment without qualifying as surrogate endpoints [4,34]. This distinction prevents the assumption that because patients with lower biomarker values have better outcomes, lowering the biomarker will necessarily improve those outcomes.

The Food and Drug Administration-National Institutes of Health Biomarkers, Endpoints, and other Tools (FDA-NIH BEST) Resource provides a standardized biomarker taxonomy according to intended role, including diagnostic, monitoring, pharmacodynamic/response, predictive, prognostic, safety, susceptibility/risk, and surrogate endpoint biomarkers [35]. Regulatory qualification programs at the FDA and European Medicines Agency (EMA) similarly assess biomarkers within a clearly defined context of use in drug development [36,37]. Regulatory qualification, however, is distinct from clinical utility in routine care, which requires evidence that biomarker-guided decisions improve outcomes or provide an acceptable balance of benefit and harm. This distinction protects against a deceptively simple assumption: if patients with lower values do better, lowering the value must make patients do better. Sometimes it does; sometimes it does not. The difference can only be established empirically.

What these examples mean for laboratory medicine

Taken together, these cases suggest a broader way of thinking about laboratory value. Analytical quality remains non-negotiable. A result must be accurate, precise, reproducible, and available at a clinically appropriate time. But these characteristics describe the quality of measurement, not the full value of testing.

Patient-centered laboratory medicine has increasingly emphasized the consequences of testing outside the laboratory itself [1]. Value-based laboratory medicine extends the same reasoning to clinical outcomes, downstream resource use, and avoidable harm [2,3].

The examples in this review show that value takes different forms. For D-dimer, the benefit is safely avoiding unnecessary imaging. For PCT and CRP, it may be reduced antibiotic exposure. For high-sensitivity troponin, it may be faster exclusion of myocardial infarction and shorter hospital stay. None of these requires a reduction in mortality to be clinically meaningful; in each case, the endpoint simply has to fit the intended use.

Population cancer screening, in contrast, demands a much higher evidentiary threshold because large numbers of healthy people are exposed to testing and its downstream consequences. Detecting additional early-stage disease is not sufficient if clinically important outcomes do not improve.

Building on the methodological frameworks of Pletcher and Pignone, Bossuyt et al., Moons et al., and others, the six case studies make the context-dependence of utility concrete as follows: the central issue is not whether a biomarker has “utility” in the abstract, but utility for what decision, in which patients, and compared with what alternative [5-8,10-12,32-34]. The principal evidence and clinical utility lesson from each case study are summarized in Table 1.

**Table 1:** Six biomarkers, six lessons in clinical utility. PCT: procalcitonin; HiSTORIC: High-Sensitivity Cardiac Troponin on Presentation to Rule Out Myocardial Infarction; High-STEACS: High-Sensitivity Troponin in the Evaluation of Patients With Acute Coronary Syndrome; MACROS2: Mersey Acute Coronary Syndrome Rule-Out Study 2; ADJUST-PE: Age-Adjusted D-Dimer Cutoff Levels to Rule Out Pulmonary Embolism; PEGeD: Pulmonary Embolism Graduated D-Dimer; IPD: Individual Patient Data; NT-proBNP: N-terminal pro-B-type natriuretic peptide; GUIDE-IT: Guiding Evidence-Based Therapy Using Biomarker-Intensified Treatment in Heart Failure; PLCO: Prostate, Lung, Colorectal, and Ovarian; UKCTOCS: UK Collaborative Trial of Ovarian Cancer Screening

Biomarkers	Illustrative evidence	Main lesson	References
D-dimer	ADJUST-PE, YEARS, PEGeD, Wells/D-dimer IPD meta-analysis	Clinical context and pretest probability can create utility	[12-15]
Procalcitonin	Randomized trials and meta-analyses of PCT-guided antibiotic discontinuation	Utility depends on the management algorithm attached to the result	[16-20]
High-sensitivity cardiac troponin	HiSTORIC, High-STEACS, MACROS2	Better detection creates interpretive responsibility; detecting more is not synonymous with benefiting more	[8,21,22]
CRP	Randomized trials, 2022 Cochrane review, recent implementation trial	Clinical value does not require technological novelty	[23-26]
NT-proBNP	GUIDE-IT and natriuretic peptide-guided therapy meta-analyses	Prognostic value alone does not validate a biomarker as a therapeutic target	[9,27,28]
CA-125	PLCO, UKCTOCS, stage-shift methodological evidence	Earlier detection or stage shift does not necessarily improve patient-important outcomes	[10,29-32]

A practical framework for evaluating a new biomarker

Evaluation of a new biomarker should begin by defining its intended population, clinical setting, decision point, comparator, and downstream action. Analytical performance, preanalytical variation, biological variability, standardization, and reproducibility must be adequate for that context. Clinical validity should then be demonstrated in the population in which the test will actually be used, rather than inferred from a selected discovery cohort.

The central assessment is whether the biomarker adds information beyond the current pathway and moves patients across a threshold that changes management. That action must be effective, and the complete testing strategy should be judged against current care using patient-important outcomes, false-positive and false-negative consequences, treatment burden, additional investigations, patient experience, and resource use. Early-stage evidence may justify further development, but it should not be described as proof of clinical utility. Seen in this way, the persistent gap between biomarker discovery and clinical adoption becomes less surprising.

Discovery research is concentrated mainly on the left side of the pathway - biological signal, measurement, association, and prediction. Clinical value is determined largely on the right - decisions, interventions, outcomes, and harms. Bridging that gap requires a different type of evidence than most discovery studies are designed to generate.

Limitations

This review has several limitations. It is a targeted narrative review rather than a systematic review, and the selected biomarkers were chosen for conceptual clarity rather than through formal ranking or exhaustive screening. This approach introduces a risk of selection and confirmation bias because examples were chosen to illustrate distinct translational patterns rather than to test the proposed framework across all biomarker classes. A broader systematic evaluation would be needed to establish how consistently the framework applies across different fields. Because this was a targeted narrative review without exhaustive study screening or diagnostic-accuracy pooling, PRISMA reporting and Quality Assessment of Diagnostic Accuracy Studies 2 (QUADAS-2) assessment were not applied.

No formal risk-of-bias tool or evidence-grading system was applied. Because this was a targeted narrative review rather than a systematic review, PRISMA reporting was not used, and QUADAS-2 and Cochrane risk-of-bias assessments were outside its predefined scope. Retrospective application of different tools to a selectively assembled and methodologically heterogeneous group of studies could give a misleading impression of systematic appraisal. We therefore do not present the included studies as having equivalent evidentiary weight. Greater emphasis was placed on prospective management studies, randomized trials, and systematic reviews, while study-specific limitations that materially affect interpretation, including early termination, heterogeneity across protocols, or failure to meet a prespecified noninferiority criterion, are described where relevant.

The case studies are drawn mainly from cardiovascular medicine, infectious disease, and cancer screening. Other contexts may require different evidentiary approaches. Pharmacogenomic biomarkers that guide drug selection, for example, may be evaluated differently from diagnostic rule-out biomarkers, while rare-disease biomarkers may face practical constraints that make large randomized outcome trials difficult.

Clinical utility is also inherently context-dependent. A biomarker that is useful for diagnosis may not be useful for treatment guidance, and performance in one population, decision threshold, comparator, or healthcare setting may not translate directly to another. The framework should therefore be applied only after explicitly defining the intended context of use, population, decision point, comparator, and downstream action.

Finally, the six case studies are established biomarkers with relatively mature evidence bases. Emerging multi-omics signatures, liquid-biopsy assays, and artificial intelligence-derived biomarkers may not yet have progressed far enough along the pathway to support outcome-based evaluation. For such markers, analytical and clinical validity remain necessary early steps, but claims of clinical utility should remain proportional to the stage of evidence available.

## Conclusions

A biomarker has true clinical utility only when its result adds meaningful information, changes a relevant decision, and leads to better care with an acceptable balance of benefit, harm, burden, and cost. Analytical validity, association, and predictive accuracy are necessary foundations, but they do not by themselves establish clinical value. Evaluation should therefore focus on the complete biomarker-enabled decision pathway, measurement, interpretation, action, and outcome, and compare that strategy with current care in the intended population and setting.
